# Yellow Jack: a modern threat to Asia-Pacific countries?

**DOI:** 10.1038/s44298-024-00079-5

**Published:** 2025-04-24

**Authors:** Duane J. Gubler, Kathryn A. Hanley, Thomas P. Monath, David M. Morens, Mauricio Lacerda Nogueira, Nikos Vasilakis, Scott C. Weaver, Yellow Fever Advisory Group, Jens H. Kuhn, Charles H. Calisher, Esper G. Kallas, Scott B. Halstead, Stephen Higgs, Laura D. Kramer, Luiz Tadeu Moraes Figueiredo, Robert B. Tesh, Andrew F. Van den Hurk

**Affiliations:** 1 Program in Emerging Infectious Diseases, Duke-NUS Medical School, Singapore, Singapore; 2 Department of Biology, New Mexico State University, Las Cruces, NM USA; 3 Quigley BioPharma, Leominster, MA USA; 4 Chester, Maryland, USA; 5 Laboratórios de Pesquisas em Virologia, Departamento de Doenças Dermatológicas, Infecciosas e Parasitárias, Faculdade de Medicina de São José do Rio Preto, São José do Rio Preto, Brazil; 6 Department of Pathology, University of Texas Medical Branch, Galveston, TX USA; 7 Center for Vector-Borne and Zoonotic Diseases, University of Texas Medical Branch, Galveston, TX USA; 8 Institute for Human Infection and Immunity, University of Texas Medical Branch, Galveston, TX USA; 9 World Reference Center for Emerging Viruses and Arboviruses, University of Texas Medical Branch, Galveston, TX USA; 10 Department of Microbiology and Immunology, University of Texas Medical Branch, Galveston, TX USA; 11 Division of Clinical Research and Integrated Research Facility at Fort Detrick, Tunnell Government Services—Contractor supporting: National Institute of Allergy and Infectious Diseases, Frederick, MD USA; 12 Department of Microbiology, Immunology and Pathology, College of Veterinary Medicine and Biomedical Sciences, Colorado State University, Fort Collins, CO USA; 13 Department of Infectious and Parasitic Diseases, School of Medicine, University of Sao Paulo, Sao Paulo, SP Brazil; 14 Department of Preventive Medicine and Biostatistics, Uniformed University of the Health Sciences, Bethesda, MD USA; 15 Department of Diagnostic Medicine & Pathobiology, Biosecurity Research Institute, Kansas State University, Manhattan, KS USA; 16 Arbovirus Laboratory, Wadsworth Center, New York State Department of Health, Albany, NY USA; 17 School of Medicine of the University of São Paulo in Ribeirão Preto, Ribeirão Preto, SP Brazil; 18 Departments of Pathology and of Microbiology & Immunology, University of Texas Medical Branch, Galveston, TX USA; 19 Public Health Virology, Public and Environmental Health and Queensland Public Health and Scientific Services, Queensland Health, Coopers Plains, QLD Australia

**Keywords:** Vaccines, Virology

## Abstract

In 1923, H.R. Carter published a seminal treatise on the possibility of yellow fever virus spreading to the Asia Pacific region, where large numbers of susceptible people were at risk of infection. This paper marks the 100th anniversary of that publication, and posits that, despite many public health advances, global trends increase the likelihood of yellow fever virus geographic spread. Potential reasons for the failure of the virus to spread are discussed.

## Introduction

Yellow fever (YF; ICD-11: 1D47^1^), an acute viral disease of humans and several species of nonhuman primates associated with high case–fatality ratios, is caused by infection with yellow fever virus^2^ (YFV; *Flaviviridae*: *Orthoflavivirus*^3^). YFV is transmitted among nonhuman primates in Africa and South America by sylvatic *Aedes* spp. and *Haemagogus* /*Sabethes* mosquitoes, respectively. While many New World nonhuman primates develop disease resembling that of humans, African nonhuman primates are generally tolerant of the infection^4^. Unlike many other mosquito-borne viruses, YFV-infected humans develop high levels of viremia and are therefore efficient amplifying hosts, capable of sustaining interhuman transmission by mosquitoes and historically giving rise to “urban” transmission cycles.

The primary urban mosquito vector is the yellow fever mosquito (*Aedes* (*Stegomyia*) *aegypti* (Linnaeus 1762)), which is highly adapted to the human environment, anthropophilic, and bolstered in urban settings by human behaviors such as peridomestic water storage. Both yellow fever mosquitoes and YFV originated in the forests of Africa and were introduced into the Americas from Western Africa via the transatlantic slave trade in or prior to the 1600 s. The ancestral, sylvatic form of the urban mosquito vector (*Ae. aegypti formosus*) adapted thousands of years ago to live in close association with people in arid regions of Africa where water storage is required and gave rise to the domesticated mosquito (*Ae. aegypti aegypti*) that is the principal epidemic vector^5^. The latter, which used shipboard containers of fresh water for its immature stages of development, readily spread throughout the western hemisphere, infesting cities and towns, primarily along the shipping and river boat commercial routes^6^.

**Fig. 1:**
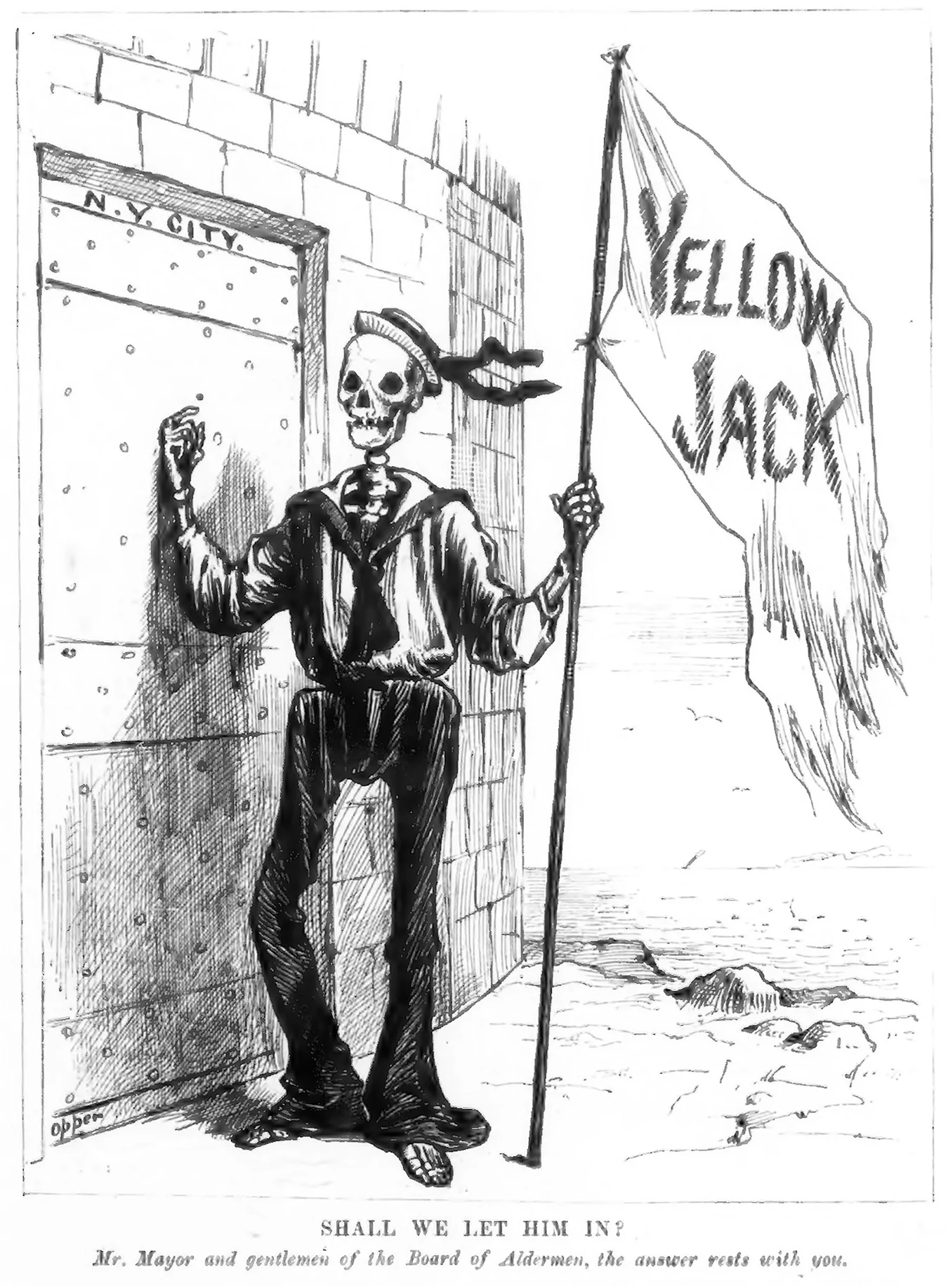
Crop of back cover image in Frank Leslie’s Illustrated Newspaper, published on September 21, 1878, in New York, NY, signed Frederick Opper (lower left). “SHALL WE LET HIM IN? Mr. Mayor and gentlemen of the Board of Aldermen, the answer rests with you.”. The figure depicts a sailor coming ashore at New York City's port with yellow fever. The caption signifies the efforts of the time to contain the import and spread of the virus. Source *Frank Leslie's Illustrated Newspaper* (New York), 21 September 1878, Vol. 47, No. 1,199, p. 48. A scan of the full page is provided as Supplementary Figure 1.

During the North American colonial era, yellow fever mosquitoes were repeatedly imported to U.S. east coast port cities by Caribbean trading vessels involved in the commerce of sugar and other goods. On-board YFV transmission could ensue, resulting in offshore quarantine when recognized by port authorities. In northeastern cities with cold winters, yellow fever mosquitoes were present only in the warmer seasons after introductions via shipping. In many Gulf Coast cities, however, they became endemic and still persist today. Once an area became infested with yellow fever mosquitoes, YFV soon followed, causing devastating YF epidemics in the 18th, 19th and early 20th centuries. During this period, YF became the scourge of the New World, with epidemics occurring as far north as Boston in the U.S., killing as many as 10% of some populations, e.g., in Philadelphia, which was the U.S. capital in 1793^7^. Some historians claim that the deadly U.S. coastal epidemics between 1793 and 98 forever changed the national character. The disease was greatly feared and was given the nickname “Yellow Jack”, referring to the flag flown on quarantined ships with onboard outbreaks (Fig. 1). In Europe, where epidemics occurred about a century after YFV introduction to the Americas, France, England, Spain and Italy suffered epidemics and YF was often referred to as the “American Plague”.

A very effective and long-lasting live-attenuated YFV vaccine, developed by Max Theiler in the 1930s, along with the near eradication of yellow fever mosquitoes in the Western Hemisphere, eventually put a stop to urban transmission of YFV in the Americas. However, enzootic cycles in nonhuman primate hosts and sylvatic mosquitoes persisted in the forest canopies of both Sub-Saharan Africa and the American tropics, the latter resulting from spillback from the human cycle centuries ago. Human-amplified urban epidemics of YF have occurred recently in Africa; however, although yellow fever mosquitoes have reinfested most of the neotropics, urban, human-amplified YF epidemics have not been documented in the Americas in over 80 years. Nonetheless, spillover from sylvatic cycles results in tens of thousands of YF cases each year. Recently (2016–23), spillover has increased in both Africa (e.g., Ghana, Cameroon, Nigeria)^8–10^ and the Americas (mainly Brazil)^11^, raising the alarm of potential spread of the YFV to non-enzootic geographic regions infested with *Ae. aegypti*^12,13^.

The reasons for the recent re-emergence of epidemic YF are not fully understood but are probably the result of a combination of factors, including: (1) expansion of human populations, (2) inadequate vaccine coverage for prevention and limited vaccine supply for emergency use during epidemics (such as in Angola and Brazil), (3) failure to enforce International Health Regulations (IHR), including the mandate that persons traveling to and from YFV-endemic areas have certificates of vaccination, and; (4) lack of effective mosquito control in urban areas. Recent, unprecedented growth of tropical cities infested with yellow fever mosquitoes, rapid and ramified international transportation, and globalization all increase the threat of YF epidemics. A 2016 study estimated that 60 million unvaccinated people traveled to or from YFV-endemic countries to areas infested with yellow fever mosquitoes^14^. Moreover, the number of confirmed, imported YF cases in nonendemic areas in 2017–2018 was the highest global figure ever recorded^12^. In 2019, it was estimated that, globally, over four billion people traveled by airplane^15^.

It is noteworthy that three other epidemic arboviral diseases, dengue, Zika, and chikungunya, also transmitted among humans by yellow fever mosquitoes, have increased in incidence and geographic footprint due to many of the same factors noted above. But, unlike dengue, Zika, and chikungunya viruses, YFV has thus far not been transmitted in Asia. However, the similar nature of the four transmission cycles suggests a clear and present danger that the geographic distribution of YFV could expand to the Asia Pacific region^13,16^. Therefore, the epidemiologic, demographic, and societal factors that influence transmission of *Aedes* mosquito-transmitted viruses in 2024 pose the greatest risk in over 70 years of YF epidemics in permissive tropical cities that have not previously experienced the disease. Of primary concern in this regard is the Asia-Pacific region, where over two billion susceptible people live in areas infested by yellow fever mosquitoes.

The perceived risk of YF expansion is not new. The possibility of YF spreading to the Asia-Pacific region was recognized in the 18^th^ and 19^th^ centuries but considered unlikely because of the logistical barrier of having to sail around the tip of South America to reach the Pacific Ocean and Asia^17^. As a result, commerce between the Caribbean region, where most YF cases were recorded, and Asia was infrequent. However, once *Ae. aegypti* was unmasked as the vector of YFV by the U.S. Army Yellow Fever Commission, the threat of YFV translocation to Asia was highlighted by Sir Patrick Manson in 1903^17^. Also in 1903, in an era in which international quarantine was among the major public health tools for controlling epidemic infectious diseases, the International Sanitary Conference in Paris^17^ adopted YF as one of five diseases requiring prevention of dispersal and recommended establishing maritime regulations, sharing disease intelligence, and quarantine. The practices were justified in 1910 and again in 1911, when cases of YF were detected aboard ships arriving in Hawai’i from the western coast of South America^18^. Fortunately, effective quarantine prevented secondary transmission in Hawai’i. With completion of the Panama Canal in 1912, the risk was thought to have increased. These events stimulated a great deal of research on the etiology, transmission, prevention, and control of YF in the first three decades of the 20th century. A fundamental question at the time was, and remains: why, in areas within the Asia-Pacific region where susceptible human populations live in crowded cities, yellow fever mosquitoes are abundant, and susceptible nonhuman primates occur at high densities even within some city centers, are YF outbreaks or even small case clusters not occurring? The extensive histories of Zika and chikungunya viruses and their introductions into Asia from their sylvatic African origins^19^ would suggest the same has probably happened with YFV. For example, during an extensive YF epidemic in Angola in 2016, there were 11 recorded instances of YF cases in workers who had returned to China from Angola, including to regions infested with *Ae. aegypti*^20^. Fortunately, no secondary transmission occurred.

Critical contributions to understanding the epidemiology of YF, virus transmission by mosquitoes, and the threat it posed to the Asia-Pacific region were made by Henry Rose Carter, an Assistant Surgeon in the Marine Hospital Service (forerunner of the U.S. Public Health Service). In the 1880s and 1890s, Carter, who was assigned to marine hospitals and quarantine positions in several southern Gulf Coast states that experienced epidemics of YF, developed a strong interest in the disease. His research, conducted in 1898, correctly showed the incubation period in humans to be less than 6 days, and that there was a “period of 10 days to 3 weeks required before successful transmission of the virus to initiate secondary human cases”^21^. This was subsequently shown by the U.S. Army Yellow Fever Commission to be the “extrinsic incubation” period in the mosquito vector. U.S. quarantine regulations and disinfection of ships arriving at U.S. ports were also based on Carter’s findings^22^.

In 1899, Carter was assigned to Cuba as Chief Quarantine Officer for the Marine Hospital Service and had numerous discussions with Carlos Finlay^23^ (who had hypothesized in 1881 that YF was mosquito-borne, leveraging earlier reports^24^) and later with members of the U.S. Army Yellow Fever Board about his (Carter’s) work showing an extrinsic incubation period that supported Finlay’s theory. Carter is rarely given credit for his seminal contributions to understanding and preventing this disease. This commentary celebrates the 100^th^ anniversary of Carter’s largely forgotten 1923 manuscript^25^, reprinted here (Supplementary information), a high-level scholarly review based on his observations of global findings regarding the threat of YF to the Asia-Pacific region at that time.

## Current risk

The intervening 100 years since Carter’s paper have seen both successes and failures in prevention and control of epidemic YF. Highlights include isolation of the virus in 1927^26^, the development in 1936 of a highly effective live-attenuated vaccine that is still widely used today^27^, eradication of yellow fever mosquitoes from Brazil in 1938 and by 1972 from 23 countries in Latin America and the Caribbean^28^, and effective prevention of epidemic transmission in the neotropics by a combination of mosquito control and vaccination and in Francophone Western Africa by vaccination. However, public health success often sows the seeds of public health failure. The success of these programs, which required sustained and costly efforts, led to complacency and lack of funding by the early 1960s, termination of most national eradication programs by the early 1970s, and, consequently, deterioration of public health infrastructure and capacity. The U.S. initiated a yellow fever mosquito eradication program in 1962 but abandoned the effort 7 years later, concluding that eradication was not feasible in the U.S. due to repeated introductions from countries that were not committed to similar policies. The persistence of these mosquitoes in limited refugia in the Americas resulted in gradual reinfestation across North and South America, which continues to the present day. The YF vaccine continued to be used for routine childhood vaccination in most endemic countries in South America, but vaccination coverage varies widely and is less than 50% in some countries^29^.

Coincident with these failures was the emergence of global trends that facilitated the re-emergence and spread of YF and other epidemic infectious diseases. These included unprecedented population growth, urbanization and creation of megacities in which millions of people live in slums that are especially conducive to *Ae. aegypti* infestation, modern rapid transportation, globalization of trade, human migration, encroachment of humans on the natural environment and of animals on the urban environment, and the global spread of yellow fever mosquitoes. Today’s megacities all have modern airports through which billions of people pass every year, many of them visiting remote locations and carrying exotic pathogens back to crowded tropical cities, where the probability of secondary transmission is increased^30^. The millions of unvaccinated people traveling to or from YF-endemic countries each year increases the probability that ultimately, YFV will be introduced into a nonendemic country infested with *Ae. aegypti*. This is underscored by the 2016 introduction of YF into China from Angola described above^31,32^. However, that episode only exemplifies the risk that such future introductions will occur, not necessarily the outcome of the next occurrence. No longer is YF occurring only in remote areas that exclude the possibility of long-distance spread. If the virus is introduced to a permissive nonendemic country, especially one located in Asia, the disease might be first misdiagnosed as dengue, likely resulting in spread before being recognized as YFV. Box 1 summarizes the most important current risk factors for epidemic YF.

There are some mitigating factors, however, that have reduced risk of YFV spread, including the Eliminate Yellow Fever Epidemics (EYE) program of the World Health Organization (WHO)^33^, which seeks to increase access to and coverage by the YF vaccine through mass campaigns and routine childhood immunization and to improve YF surveillance in Sub-Saharan African countries. In concert with the EYE program, manufacturers have increased the annual supply of vaccine. The WHO maintains an emergency stockpile of YF vaccine, and clinical trials have shown that 17D vaccine is effective when given at one-fifth or one-half dose^34,35^, stretching the available supplies in an emergency. In addition, new approaches to controlling yellow fever mosquitoes and reducing their vector competence using mosquitoes infected with *Wolbachia* endosymbionts^36,37^ are also showing promise for the control of dengue, and, by extension, YF^38^.

Another mitigating factor is that severe YF is a striking clinical disease. Although YF may be misdiagnosed in its early stages or in milder cases, the severe form of the disease (with jaundice and hemorrhagic diathesis) is quite easily diagnosed. Travel history would raise the index of suspicion of YF further and it is likely that such imported cases would be recognized fairly early, so that isolation in hospitals and other control measures could be instituted. Factors that might delay recognition include the high rate of mild illness associated with YFV infection, and cross-orthoflavivirus immunity that complicates serodiagnosis. Studies in nonhuman primates with prior dengue or Zika virus infection appear to show reduced severity of YF^39,40^.

Unfortunately, most countries infested with yellow fever mosquitoes do not have the public health infrastructure to effectively control the mosquitoes, distribute the vaccine when it becomes available, or triage the large number of clinical cases that would inundate hospitals. Thus, a large YF epidemic would create chaos in a nonendemic country because it is a hemorrhagic disease with a high case–fatality ratio and because vaccine supply, clinical support, and mosquito control are inadequate and cannot be upscaled at the pace needed to contain epidemic transmission. The world is clearly at high risk for epidemic/pandemic YF^12^. The recent YF epidemics in Angola and Brazil demonstrated the limitations of massive emergency immunization drives, fueled severe global shortages of YF vaccines, and led to the implementation of a fractional-dosing strategy to provide vaccination coverage^34,41^.

Box 1 Risk factors for urban epidemics of yellow fever (YF) today?

1. Unprecedented urban growth
2. Large crowded tropical urban centers that provide ideal ecological conditions to maintain viruses and mosquito vectors
3. YF-enzootic areas of tropical Africa and South America with mobile populations are increasingly fragmented and accessible
4. Modern transportation provides ideal mechanisms to move viruses and vectors among permissive population centers
5. Yellow fever mosquitoes have achieved global distribution in the tropics and subtropics and continue to expand geographically
6. Yellow fever mosquitoes are evolving resistance to most standard insecticides
7. Mosquito control has been ineffective in preventing epidemics of other *Aedes* mosquito-transmitted viruses such as dengue, chikungunya, and Zika viruses
8. Sylvatic cycles of yellow fever virus (YFV) established in Africa and South America are impervious to vector control
9. The global at-risk susceptible population exceeds 3.6 billion people
10. International Health Regulations (IHR) proof of YF vaccination is not enforced
11. Low herd immunity in humans living in permissive countries in Asia and many parts of the Americas
12. Increased encroachment of humans on sylvatic YFV cycles and of YFV from sylvatic cycles on urban areas, resulting in increased contact with nonhuman primates in Sub-Saharan Africa and South America
13. YF vaccination as an emergency response tool to control epidemic spread of YF has been constrained by vaccine shortages and logistical limitations for rapid vaccine coverage
14. Inadequate YF vaccine manufacturing capacity
15. Egg-based production of YF vaccine makes it more difficult to scale up supply quickly
16. Vaccine hesitancy and misinformation about vaccines has increased in recent years

## Epidemic spread

The numerous risk factors noted above raise the question of why a YF epidemic hasn’t already occurred in permissive urban centers of tropical America or the Asia Pacific (Carter’s paramount question over a century ago, when the global population was a fraction of what it is today and before international travel had been revolutionized by airplanes)? The recent pandemics of dengue, chikungunya^42,43^, and Zika virus disease^44^ underscore that the epidemiological conditions appear to be ideal for regional and global transmission and spread of YFV, which has the same transmission requirements in tropical urban centers. Unraveling this enigma—why YF has not spread to Asia, whereas other *Ae. aegypti-*transmitted diseases have—can help us develop effective measures to prevent the spread of YF as well as other *Aedes*-transmitted viral diseases.

There are a number of possible explanations for lack of YF spread, listed in Box 2. While all may contribute at least partially to containment of YF, it is likely that their contribution is variable, depending on time and place. Also, some likely play a more important role. In particular, most (or perhaps all?) permissive areas in tropical Africa, the Americas, and Asia are endemic for dengue viruses and other orthoflaviviruses related to YFV, although seropositivity varies widely. If cross-reactive immunity mitigates clinical disease, increasing the risk of YFV infection being misdiagnosed as dengue will increase^40^. If cross-reactive immunity suppresses transmission, then YFV establishment may be less likely. First, a study in Ecuador reported that dengue immunity reduced the severity of human YF^45^. Cross-reactive immunity among orthoflaviviruses is complex, sometimes asymmetric, and difficult to infer from animal models^39,46–51^. Second, a study of YFV infection of dengue virus-immune and naïve monkeys resulted in lower viremia and milder disease in dengue immune compared to naïve monkeys^39,40^. Similar observations have been reported in nonhuman primates immune to Zika virus and exposed to Wesselsbron virus^52^. This orthoflavivirus cross-reactive immunity did not protect nonhuman primates against YFV infection, but immunity to dengue or Zika viruses nearly completely eliminated YFV infection of yellow fever mosquitoes feeding on macaques during peak viremia^40^. Thus, the increase in dengue hyperendemicity in South America and Asia may be reducing the availability of efficient human amplification hosts through cross-reactive immunity. However, this hypothesis must be confirmed in humans.

Longitudinal YFV maintenance in nature depends on sylvatic circulation of the virus in nonhuman primate hosts and mosquito vectors adapted to feed upon them. Modeling studies in South America have identified nonhuman primate population dynamics, immunity, and infection prevalence as key components in predicting spillover events to humans. The nonhuman-primate-mosquito cycle is established in the neotropics and Sub-Saharan Africa, but it is uncertain which vectors and hosts, if any, could fulfill that role in Asia^53^. Moreover, many of the most prevalent nonhuman primates in Asia are macaques, in which YFV infection results in high viremia followed by rapid (generally ≤6 days) death^54^. It is possible that the brevity of the transmission window could truncate forward transmission of the virus^55^, but if so, these dynamics did not prevent establishment of a sylvatic YFV cycle in neotropical primates, many of which also rapidly succumb to YF^56^. Intriguingly, experimental studies have also shown that macaques generated a robust and durable neutralizing antibody response against YFV following infection, while the response of sooty mangabeys, an African host of the virus, was transient^57^. Whether re-infection of African reservoir hosts of YFV contributes to its maintenance should be further explored.

Another critically important but poorly understood factor is the influence of viral genetics on transmission dynamics. Experimental infection studies have shown differences in susceptibility of nonhuman primates to different YFV isolates^58^. In the neotropics, YFV has, to a large extent, been confined for over 80 years to a sylvatic cycle involving nonhuman primates and *Haemagogus* and *Sabethes* mosquitoes. It is possible that this isolation has resulted in the virus losing its ability for efficient transmission in the yellow fever mosquito-human urban cycle. However, some experimental work has contradicted this hypothesis^59^. More research is needed to clarify all of the potential issues raised here.

Finally, there are limited data on the force of infection, determined principally by the kinetics of YF viremia in humans, including both during asymptomatic infections and at various stages of the disease, the relationship of viremia and clinical signs as well as the duration of effective viremia (i.e., capable of infecting mosquitoes). Differences in the kinetics of viremia among YF, and dengue, Zika, and chikungunya viruses could well explain the potential for secondary transmission and epidemic spread. This could also be influenced by the competence of urban mosquito populations. It has recently been shown that *Ae. aegypti* populations from Singapore, Taiwan, Thailand, and New Caledonia are capable of transmitting YFV in the laboratory^60^. However an earlier study showed considerable variation in susceptibility to YFV in Asian and American populations of this mosquito^61^. More research on these critical features of YF infection is urgently needed.

Box 2 Potential reasons why epidemic YF has not occurred in urban centers of South America and Asia

1. Cross protective immunity from dengue, Zika, and other orthoflavivirus infections
2. No YFV lineage adapted to the yellow fever mosquito-human urban cycle
3. Barriers of YF immunity in ecological edge areas
4. Geographic and demographic obstacles
5. Dynamic nature of sylvatic foci
6. Uncertain elements that would support a sylvatic cycle in Asia
7. Variable yellow fever mosquito densities and vector and host competence
8. Acutely ill YF patients have less exposure to mosquitoes
9. Good surveillance and rapid response containment
10. Effective mosquito control in areas at risk
11. Plain old luck

## Preventing pandemic YF

Effective prevention and control of epidemic YF will require a better understanding of the biology, genetic diversity and epidemiology of YFV and its mosquito vectors as well as improved surveillance and better tools to detect, contain and prevent transmission. A safe and highly effective YF vaccine is available, but it is in limited supply and would require significant upscaling for more widespread global use. While new mosquito control tools are in the pipeline and look promising, none yet provides effective emergency or long-term sustainable control at the needed scale. Surveillance for YF is generally poor in endemic countries and non-existent in non-endemic countries. In addition, public health infrastructure must be built to deal with *Aedes*-transmitted viral diseases. Therefore, successful YF containment will require extensive research and capacity-building in most tropical urban centers. Important research questions are listed in Box 3.

Box 3 Research needed to help prevent YFV epidemic spread

1. Transition from egg-based YF vaccine to more advanced and/or diverse production platforms with potential for rapid expanded supply
2. More effective sustainable mosquito control tools
3. Strategies to integrate vaccination and mosquito control
4. Antivirals to prevent and/or treat severe YF disease
5. Proactive surveillance systems
6. Role of orthoflavivirus cross-reactive immunity in transmission dynamics and clinical presentation
7. Role of YFV genetics in determining viral virulence, fitness, and transmission dynamics
8. Urban renewal to decrease ecological conditions that attract yellow fever mosquitoes

## Conclusions

The concern that YFV will spread to the Asia Pacific region that Carter raised 100 years ago in his treatise on the subject remains equally pressing today. Indeed, we would argue that the likelihood of such a catastrophe could be even higher now than it was in Carter’s time. The risk in 2025 is aggravated by ideal epidemiological/ecological/social conditions for transmission and spread of the virus and its mosquito vector. A YF pandemic in today’s world would cause a devastating public health crisis that, because of the much higher lethality, would make the COVID-19 pandemic pale by comparison. It is unlikely that the global trends (population growth/migration, urban growth, and modern transportation) responsible for this increased risk will abate in the near future.

## Supplementary information

Supplementary Information

Supplementary Figure 1

## Data Availability

No datasets were generated or analyzed during the current study.
