# Publisher Correction: Yellow Jack: a modern threat to Asia-Pacific countries?

**DOI:** 10.1038/s44298-026-00247-9

**Published:** 2026-10-09

**Authors:** Duane J. Gubler, Kathryn A. Hanley, Thomas P. Monath, David M. Morens, Mauricio Lacerda Nogueira, Nikos Vasilakis, Scott C. Weaver, Yellow Fever Advisory Group, Jens H. Kuhn, Charles H. Calisher, Esper G. Kallas, Scott B. Halstead, Stephen Higgs, Laura D. Kramer, Luiz Tadeu Moraes Figueiredo, Robert B. Tesh, Andrew F. Van den Hurk

**Affiliations:** 1 Program in Emerging Infectious Diseases, Duke-NUS Medical School, Singapore, Singapore; 2 Department of Biology, New Mexico State University, Las Cruces, NM USA; 3 Quigley BioPharma, Leominster, MA USA; 4 Chester, Maryland USA; 5 Laboratórios de Pesquisas em Virologia, Departamento de Doenças Derma tológicas, Infecciosas e Parasitárias, Faculdade de Medicina de São José do Rio Preto, São José do Rio Preto, Brazil; 6 Department of Pathology, University of Texas Medical Branch, Galveston, TX USA; 7 Centerfor Vector-Borne and Zoonotic Diseases, University of Texas Medical Branch, Galveston, TX USA; 8 Institute for Human Infection and Immunity, University of Texas Medical Branch, Galveston, TX USA; 9 World Reference Center for Emerging Viruses and Arboviruses, University of Texas Medical Branch, Galveston, TX USA; 10 Department of Microbiology and Immunology, University of Texas Medical Branch, Galveston, TX USA; 11 Division of Clinical Research and Integrated Research Facility at Fort Detrick, Tunnell Government Services—Contractor supporting: National Institute of Allergy and Infectious Diseases, Frederick, MD USA; 12 Department of Microbiology, Immunology and Pathology, College of Veterinary Medicine and Biomedical Sciences, Colorado State University, Fort Collins, CO USA; 13 Department of Infectious and Parasitic Diseases, School of Medicine, University of Sao Paulo, Sao Paulo, SP Brazil; 14 Department of Preventive Medicine and Biostatistics, Uniformed University of the Health Sciences, Bethesda, MD USA; 15 Department of Diagnostic Medicine & Pathobiology, Biosecurity Research Institute, Kansas State University, Manhattan, KS USA; 16 Arbovirus Laboratory,Wadsworth Center, New York State Departmentof Health, Albany, NY USA; 17 School of Medicine of the University of São Paulo in Ribeirão Preto, Ribeirão Preto, SP Brazil; 18 Departments of Pathology and of Microbiology & Immunology, University of Texas Medical Branch, Galveston, TX USA; 19 Public Health Virology, Public and Environmental Health and Queensland Public Health and Scientific Services, Queensland Health, Coopers Plains, QLD Australia

Correction to: *npj Viruses* https://doi.org/10.1038/s44298-024-00079-5, published online 24 April 2025

In the original version of this Perspective, an image that was not an original front-cover cartoon published in the 19th century was used as Fig 1. The image used was a composite mash-up, possibly created with AI, with no historical authenticity. The correct image has now replaced it. This editorial cartoon was printed on a small portion of the back page of the issue. The cartoon is signed Frederick Opper (lower left), “Yellow Jack. Shall we let him in? Mr. Mayor and the gentlemen of the Board of Aldermen, the answer rests with you,” *Frank Leslie’s Illustrated Newspaper* (New York), 21 September 1878, Vol. 47, No. 1,199, p. 48.

The authors are grateful to Bert Hansen of Baruch College, CUNY, USA, for bringing the error to their attention.

New figure legend:

Crop of back cover image in Frank Leslie’s Illustrated Newspaper, published on September 21, 1878, in New York, NY, signed Frederick Opper (lower left). The caption read “SHALL WE LET HIM IN? Mr. Mayor and gentlemen of the Board of Aldermen, the answer rests with you.”. The figure depicts a sailor coming ashore at New York City’s port with yellow fever. The caption signifies the efforts of the time to contain the import and spread of the virus. Source *Frank Leslie’s Illustrated Newspaper* (New York), 21 September 1878, Vol. 47, No. 1,199, p. 48. A scan of the full page is provided as Supplementary Figure 1.

