# Supplementary material for: Yellow Jack: a modern threat to Asia-Pacific countries?
Source: Npj Viruses. 2025 Apr 24;3:34. doi: 10.1038/s44298-024-00079-5 (PMC12022128; doi:10.1038/s44298-024-00079-5)
Supplement: Supplementary file 1 — Supplementary Information [file 44298_2024_79_MOESM1_ESM.pdf]

SECTION IX.—HYGIENE.

CHAIRMEN.—MELBOURNE MEETING: DR. R. R. SAYERS, *U.S. Bureau of Mines.*
SYDNEY MEETING: DR. C. J. MARTIN, M.D., D.Sc., F.R.S., *Lister Institute, London.*

A.—MELBOURNE MEETING.

1. The Chance of the Extension of Yellow Fever to Asia and Australasia.

By Dr. H. R. Carter, Assist. Surgeon-General, U.S. Public Health Service (retired).

The tendency of civilization is to disseminate the infections of man. The same is true, indeed, of those to which the animals, and even the plants associated with him, are liable. This is to be balanced, and distinctly on the debit side of civilization, against the dissemination and improvement of useful plants and domestic animals.

There are, however, a certain number of infections pathogenic to man which are not yet of universal or, indeed, of general distribution even among civilized men. In some, due doubtless to inability to sustain themselves when introduced into new environments and in some from never having been introduced. Among these we may instance both the African and American trypanosomiases; the American leishmaniasis (espundia); the verruga of Peru; certain forms of schistosomiasis and yellow fever. For most of them it would probably be necessary to introduce, not only the causative organisms, but their living vectors, and for some possibly (for perpetuity) their reservoir hosts as well.

For yellow fever, however, there is a large part of the globe yet free therefrom in which all the conditions are such that were this infection —i.e., its causative organism—introduced, it would, if left to nature, propagate itself and continue indefinitely. Of the conditions requisite for the propagation of some of the infections named above as still limited locally, we may not be able to speak confidently, but for yellow fever we have this knowledge, and it is definite: Yellow fever will propagate and continue in any community if these three factors co-exist therein—(1) the causative organism of the disease; (2) functionally active *Aëdes (Stegomyia) ægypti* mosquitoes; and (3) men susceptible

to yellow fever, the insects having free and continued access to both of the other two. The causative organism may be (*a*) in men infected with yellow fever, or (*b*) in the infected mosquitoes. These conditions are sufficient for the propagation and continuance of yellow fever. Where they exist to a sufficient extent yellow fever will spread and continue. It is fair to say, I think, that in practically all parts of the Americas wherever the temperature and sociological conditions have allowed the second factor for the propagation of yellow fever to exist, the parasite has at one time or another been present (whether introduced or indigenous I do not say), and yellow fever has prevailed, and in some places is still prevailing.

Not so for the Old World. There is a large part of it—large in area and very large in population—in which the (known) insect vector, *Aëdes* (*Stegomyia*) *ægypti*, is abundant and active, and in which yellow fever has never prevailed. In this section is comprised much of East Africa; south and south-east Asia (including southern Japan; the Philippines; the Straits Settlements and Dutch East Indies); Australia, except the extreme south; and a number (nearly all) of the islands of the Pacific and Indian Oceans. Taylor (1917-19); Edwards (1919); McFie and Ingram (1921).

Naturally, since yellow fever has not prevailed in this area, men susceptible to yellow fever, the third requisite for its propagation, are also abundant in it—more abundant and more densely grouped in some parts of it than anywhere else on earth—and, were the causative organism introduced, the infection could scarcely fail to propagate. In a large part of this area, too, the temperature is such that *ægypti* would be active all the year round, and susceptible men in these parts being also very abundant, the infection would, unless prevented by sanitary measures in them, be permanently endemic. Australia, Japan, the Straits Settlements and some other countries could, and doubtless would, protect themselves, and by sanitary measures eliminate the infection, if introduced, but considering the sociological condition of much of this *ægypti*-infested region, one could not expect this to be done by all, or indeed the most of it. There would thus result, not only an almost unspeakable calamity to much of the region specified, but a continual threat of yellow fever to all other countries in the zone of permanent *ægypti* infestation—almost the whole tropical and sub-tropical world. This would involve in such countries a great increase in either (*a*) measures to prevent the introduction of yellow fever—maritime quarantine with its restrictions on commerce—or (*b*) measures in control of *ægypti* production in such countries so as to render them uninfectable to yellow fever if introduced. Either one is costly; either one is troublesome; either one would have to be continued indefinitely, and the first, indeed, long continued does not give absolute security—"a sieve not a dam."

Moreover, the measures for the control of the vector in the Old World (should I include your new settled continent in the Old World?) may be more difficult than they have proved in the Americas—not only sociological, but from biological reasons. I think we can say that the three conditions given on page 1377 as sufficient for the propagation and continuance of yellow fever in any community are, in the Americas, also necessary therefor. It may be somewhat different elsewhere. We feel satisfied—mainly from epidemiological studies—that the mosquito named is, in nature, the only vector in the Americas of this infection to man. For that part of the Old World which I have indicated this may not be true. From analogy with malaria, trypanosomiasis, and a number of other host-borne infections one would expect that some other mosquito of the same genus and sub-genus as the one we have incriminated would also be efficient as a vector, and that none of a different genus or sub-genus would be. Analogy, of course, is unsafe to depend on, but is good as a guide of the direction investigation should take.

Now I think that practically all the investigations on which we depend for our knowledge of the mosquito conveyance of yellow fever have been made in the Americas, and, naturally, involve only the American mosquitoes. In the Americas we have no species of this sub-genus, *Stegomyia*, except *ægypti*. Obviously, American observations—negative for American mosquitoes, except *ægypti*—could not consider the other species of this sub-genus, among which one would naturally expect to find vectors, and it may well be that some other species of *Stegomyia*, of which there are a large number in the Old World, may also be vectors of yellow fever! If so, the problem of control of the vector—since the breeding habits of the different species of *Stegomyia* are not quite the same—would be decidedly more difficult in the Old World than it is with us—and with us it has not always been easy.

In the spring of 1914, the writer suggested to Sir Wm. Leishman, then a member of the British West African Yellow Fever Commission, that experiments be made to determine if *Aëdes* (*Stegomyia*) *scutellaris*, now known as *alba picta*, was a vector of yellow fever. This species, common in West Africa, Australasia, south-east Asia, and this region generally, is quite like *ægypti* in its biology, but apparently less differentiated as a co-mensual with man, and certain measures would be needed to control its breeding in addition to those sufficient for *aegypti*.

What is the risk and whence is the threat of the introduction of yellow fever into the *ægypti*-infested region we have specified? The direct risk to any part of these regions is in some measure proportionate to (1) proximity (in time) to ports infected with yellow fever; (2) the amount and nature of the commerce between them, and (3) the

sanitary measures taken with this commerce (*a*) in the infected ports—to prevent infection aboard the vessels; (*b*) on leaving or *en route* therefrom—to clear them of any received—and (*c*) on arrival at the port of entry—to prevent the introduction of any infection still existing aboard to such a port. We must note, too, that the direct risk may be different from the indirect, and that the risk in some future contingency may be entirely different from what it is now.

Of this I think we may say, too, that Australia is at present little threatened directly. It is very distant from and, so far as I can find, has little direct communication with, ports infected with yellow fever. Nevertheless, were yellow fever introduced into South-east and South Asia and the adjacent islands, the infection would quite certainly be permanent and their infection would be a very grave risk to Australia. The danger of infecting these regions then must be considered as a grave, if indirect, danger to Australia itself—a stepping stone for the infection and with almost a certainty of it, some time, taking the second step.

The threat is naturally from the places where yellow fever prevails, or, indirectly, is likely to prevail. The only regions in which yellow fever now exists are tropical America and West Africa. Its introduction then into the regions we are considering (except into East Africa) can only be by vessel; hence the word "port" is used above instead of "place."

Mechanism of Introduction of Yellow Fever. It can be introduced by two agencies—(1) infected men, and (2) infected mosquitoes. The causative organism has no free living stage, and we know of no host for it of sanitary importance other than man and the mosquito. Either host may come aboard infected, one of them must, or be infected aboard—men by infected mosquitoes, mosquitoes by infected men.

(1) Men coming aboard infected at any port at which yellow fever now exists are not a direct source of danger—*i.e.*, would not themselves convey infection—to Asia. The time between the closest suspected part and Asia is well over that from the beginning of infection of man to the termination of his infectivity to the mosquito—say, fifteen days. (The sum of the maxima of the hitherto observed incubation in man, plus his infectivity to *ægypti*, is 6 1/12 + 3—ie., 9 1/12 days). He would, however, be an indirect and very serious danger by infecting *ægypti*, if the vessel carried them. Men infected aboard imply *ægypti* aboard, and would be a source of danger (*a*) directly, by landing while infected, either sick or (and worse) in the stage of incubation, depending on how long before arrival they were infected, and (*b*) indirectly, by infecting other mosquitoes aboard. The presence of yellow fever in men on arrival or the history of any fever, unless clearly not yellow fever, *en route* from an infected port is such a

warning that the receiving port would naturally safeguard itself, and thus should enormously lessen the risk of its introduction. The risk is where infection is not suspected.

(2) The insect host, whether infected aboard, or received aboard already infected, is far more dangerous than the human. It retains infection and the power of infecting men for a long time—57 days has been proven, and probably as long as they live. And Fielding (1919-20) in Australia has kept one alive for 91 days.

Moreover, unless they infect some man, and do so some days before arrival, they give no evidence of infection aboard the ship which would warn the port authorities to take extra precautions. The experiments of Flu (1920) show that *ægypti* imagos do not survive six days in the refrigerator compartments (for fruit) of a vessel kept at about (a little above) 6° C. (43° F.). All die in 24 hours if the temperature is not over 6° C. It is, then, not possible to transport infected insects any distance, in time, in refrigerated compartments, either for fruit or for meat. Nevertheless, it is possible that, confined to some other part of the vessel not visited by men, they might infect no one *en route*. Such cases, while not at all common, have occurred. It is, then, of prime importance (*a*) to prevent infected mosquitoes coming aboard, and (*b*) scarcely less so—although less so—to keep all *ægypti* (indeed all *Stegomyia*) from coming aboard, or, and especially, breeding aboard, as, without them, men infected with yellow fever may be received aboard without risk. The danger of conveying yellow fever to a distant place (meaning distant in time) centres around the insect vector.

RISKS FROM THE AMERICAS.

West Coast of America. We have said yellow fever exists in tropical America. Ships can go direct from the warm ports of the Pacific coast of America direct to Asia, keeping the whole route in weather warm enough for *ægypti* imagos to live and to function. From such ports, if infected, there would be a direct risk, the degree depending on the factors already given of introducing yellow fever into Asia.

East Coast of America. There are two routes into the Pacific and to Asia: one via the Straits of Magellan (or for sailing vessels around the Horn), and the other by the Panama Canal. For the first vessel, even a steamship, must stay so long in cold weather that *ægypti* imagos, unless kept alive by artificial heat, die, and the time she must spend in temperature below the feeding limit of this species (62°-64° F.) is so long that cases of yellow fever in men contracted in warm weather would be well past the stage infective to mosquitoes before any of the species, surviving in the egg stage, were again able to feed on men. Note here that breeding of this species, while common aboard sailing vessels, exposed on this trip to longer and greater cold,

is rather rare on steamships. Vessels taking this route are quite certainly then free from infection when they reach any infectable port on the Pacific. It is true that imagos could be kept alive in artificially-heated compartments. Nevertheless, no infection has ever been reported as carried by this route to any Pacific port of South America, or developing on any vessel which has made this passage. We may consider this route, I think, then, as safe.

The route through the Panama Canal, like that from the warm Pacific ports of America, can be made direct to Asia entirely in warm weather, and would, except for the added time consumed, involve the same direct risk to that country were no sanitary measures taken. Every vessel, however, which enters the Panama Canal ("infected or suspected of infection with yellow fever") is subjected to measures of sanitation: (1) all mosquitoes aboard are killed. Imagos by a gaseous culicide—generally hydrocyanic gas—and their breeding places destroyed, (2) the personnel, crew and passengers, are held six days—from their last exposure to possible infection, *i.e.*, the fumigation of the ship—to cover the period of incubation of yellow fever, so that, if any one does develop it, this will be at the Quarantine Station, where there are no mosquitoes. The vessel then leaves the Canal free from infection and free from mosquitoes.

Not much chance is taken. That the vessel is from a port "in which yellow fever exists, or is suspected to exist," places her at once in the category of a "suspected" vessel, unless the Quarantine Officer of the Canal is satisfied—(*a*) that she received no infection at that port, or (*b*) was freed from infection on or after leaving; both depending on the sanitary measures taken. No vessel passing the Canal has so far ever developed yellow fever aboard, or been accredited with carrying infection elsewhere.

I think, then, we may claim that yellow fever confined to the east coast of America involves practically no risk to Asia directly, or indirectly by infecting the Pacific Coast of South America. These measures are especially designed for the protection—(*a*) of the West Coast of South America, and (*b*) for the far East. Extremely few vessels take this trip from a tropical port east of the Canal to one on the Pacific.

YELLOW FEVER IN AMERICA.

We count three foci of yellow fever in the Americas:—(1) the northern coast of Brazil, (2) the northern part of South America south of the Caribbean Sea, and (3) in Mexico and Central America.

(1) The Brazilian infection is on the northern coast. Yellow fever has not been reported south of Victoria for many years—say since 1908—nor in Para, the principal port of the north, since about 1911. It

was eliminated from Rio Santas and Para by sanitary measures—prevention of *ægypti* production—and there is no reason to believe that these measures will be relaxed so as to again allow of the infection of the ports thus freed from it. Indeed, there is much more reason to believe that this work will be extended, and the whole littoral—and ultimately the whole of Brazil—freed from this infection. Commerce from this region might carry yellow fever directly to Porto Rico, to some other ports of the United States of America, Trinidad, and possibly Barbadoes, although the latter is not on the itinerary of regular lines north-bound from the infected ports. There is also direct trade with Europe and with West Africa. The first, mainly to non-infectable territory, is of no consequence to us; the second is already infected.

The risk of infection to Porto Rico or any port of the United States of America with the system of maritime quarantine prevailing is slight; the chance of their remaining infected beyond a short time, with the sanitary measures which would be applied, practically nil. There is no direct trade with Mexico or Central America, or, except an occasional call at Port of Spain, with the north coast of South America. For reasons already given, this Brazilian focus involves no risk to Asia, either directly or by the infection of the Pacific Coast of South America.*

(2) The existence of yellow fever at Bucaramanga—a town of 30,000 in the north-eastern mountains of Colombia—is a great disappointment. It is probably the remains—and it may be not all of the remains—of the great regional focus of the "South Caribbean and adjacent islands," which extended on the mainland from Panama to the Guianas, with the islands along the coast, and the Lesser Antilles. This was for a long time a permanent endemic regional focus, and an important one. After, and influenced by, the sanitation of Panama, yellow fever spontaneously disappeared from a great part of it. Indeed, we had hoped from all of it; none had been reported in it since 1917—at Coro, Venezuela, by Guiteras—until a few days ago. We do not yet know the extent of the infection in this region. Should it reach the coast, as is probable, it will be a source of danger to Mexico and Central America when (if) they are freed from infection, and thus be an indirect risk to Asia through the Pacific ports of these countries.

(3) Yellow fever has existed in Mexico or Central America, counting them as one regional focus—sometimes in one part, and sometimes in another, and it may be with intervals when no part of either was infected—for a very long time. We can certainly say, noting the above proviso, since 1648.

* There is no direct trade between this part of Brazil with Mexico and Central America, but should, as we think, Mexico and Central America be freed from yellow fever before Brazil, there is always a possibility of the latter infecting them indirectly through some other country and thus again involving the Pacific ports of these countries. Slight as this risk appears, we shall be glad when it is eliminated. Should Brazil remain infected indefinitely there would be ultimately grave risk (almost a certainty) of infection of the Pacific coast of South America by way of the upper Amazon, as travel routes will ultimately be opened across the Cordilleras. This is not imminent in the near future, however.

This focus may be no longer existent. A few years ago a generalized epidemic of yellow fever spread over practically all of the infectable (*ægypti*-producing) part of Mexico and Central America, consequent on the political disturbances in the former country. A campaign against this was undertaken in 1921 by the International Health Board of the Rockefeller Foundation, and it may be that at this writing (23rd May, 1923) this infection is eliminated from this focus and from North America. We cannot be sure of this yet, however, and the work against the insect vector is still going on, and it is purposed to continue it until this elimination is certain.

There is quite certainly no yellow fever anywhere in the Americas outside of these three regional foci. The permanently endemic regional focus of the West India Islands is extinct. The same is true of that so long on the Pacific Coast of South America, of which Guayaquil and Panama were the infecting centres. Moreover, the trade relations are such that, as long as they continue as at present, the risk of this coast again receiving infection is minimal, no vessel coming to it from any port infected with yellow fever.

There is, indeed, no infection at present in any of the Pacific ports of the Americas (the only ones which involve direct risk to Asia)—not even in those of Mexico and Central America. Until, however, we can say that these countries (our third regional focus) are free from infection, we cannot be sure that these ports will not be reinfected. They were infected two years ago. Nor even if freed from infection is this region entirely safe from re-infection from the focus south of the Caribbean of which we have spoken.

There is, then, at present no direct risk of the introduction of yellow fever into Asia from America, and the only indirect risk comes from the chance of the re-infection of the Pacific ports of Mexico and Central America.

TRANS-PACIFIC COMMERCE FROM THE WEST COAST OF THE AMERICAS.

The time at the writer's disposal has not been sufficient for a proper investigation of this commerce. We are quite sure that none of the Pacific ports of the Americas are at present infected, those north of Mexico are uninfectable, the coast from Panama south is little liable to infection. Only those of Central America and Mexico, then, need be considered as, while not infected, they must be held liable to infection until yellow fever is eliminated from that great regional focus.

There are several regular lines and a number of tramp vessels going through the Canal and across the Pacific from Europe and the Atlantic ports of North America. These, of course, we need not consider. There is at present no line, and very rarely even a tramp steamer, touching the tropical ports of America so doing. The only lines of which I can hear that touch at any Pacific port of America south of California

and across the Pacific are—(1) the Toyo Kisen Kaisha of Japan, an old and well established line; (2) the Hwah Ping of China; and (3) the Vier, India-Chilean Line.

The first makes from six to nine trips per annum between the Pacific ports of the Americas and Asia. It carries both freight and passengers, the latter in considerable number—first, second, and third class, 200 to 250 at times. The regular itinerary of the return trip is from Valparaiso—some of the nitrate ports of Chile, Callao, Los Angeles, San Francisco, Honolulu, Japanese ports, Hong Kong, and Shanghai. Salina Cruz, Acapulco, and Manzanillo, all Mexican, are on its list of ports of call as optional, but the San Francisco office now gives this line as touching at no port between Callao and Los Angeles, California, and I am informed that there is now no Japanese Consular representative on the west coast of Mexico. They have, however, within the past three years touched at Manzanillo and at Balboa, Canal Zone. From Manzanillo to the first Asian port is about six weeks; to Honolulu about two weeks—a little over. Honolulu is naturally a safeguard to the Asian ports, as a complete system of maritime quarantine is maintained there. As, naturally, being closer, it is more exposed to infection from the Americas than Asia, and were it allowed to become and to remain infected, would be a convenient stepping stone for yellow fever to reach Japan.

The second is a single ship—the *Hwah Ping*—belonging to the Chinese Government. She made two trips within the past year between China and the Pacific ports of the Americas. Her itinerary was much like the Japanese line, but touched at Manzanillo. Whether this will develop into a regular line is uncertain. My informant thinks not.

(3) The Vier Line makes three trips per annum from Calcutta, Colombo, Valparaiso, and Callao, via Australia. It touches no port at which yellow fever has prevailed for a number of years, or at which it is likely to prevail.

The amount of this traffic is thus very small.

Of its nature there is this to say: Neither the Vier nor the Japanese line of last year involved any risk whatever, visiting no ports infected or liable to be infected. The Chinese vessel visited no infected port—there was none on the Pacific—but did visit some liable to infection, in that we cannot at present guarantee that Mexico is free from yellow fever. One will feel safe when yellow fever is definitely eliminated from Mexico and Central America, and to that end we are working.

Of the sanitary measures taken to prevent the introduction of infection aboard vessels engaged in this commerce, to eliminate it if introduced, and to prevent its transference to the Asian ports of entry, direct knowledge is almost entirely lacking to the writer. There is little need for such measures as long as the west coast of America keeps

in its present sanitary condition. In addition, wharfs are conspicuous by their absence in the tropical ports of America—except at Balbao—and the vessels lie far off shore, so the chance of getting infected *ægypti* aboard, should any of these ports become infected, is not great. The Japanese line has been found, in general, not breeding *ægypti* abroad at the Honolulu Quarantine Station, and, from what the writer knows of the handling of the Manila-San Francisco vessels—when there was cholera in Manila—the inspection and other quarantine measures at the Japanese quarantine station are careful and scientific.

That the *Hwah Ping* is the property of the Chinese Government is, possibly, a danger, as it is a question whether she would be subjected at a Chinese port to the same sanitary restrictions as a vessel privately owned.

YELLOW FEVER IN AFRICA.

The writer knows nothing of Africa at first hand. He can only judge of the conditions there from the reports (verbal and written) of others. Yet as this study would be otherwise entirely incomplete he is submitting deductions from such knowledge as he has of the yellow fever in Africa. He believes these deductions are justified as a conservative interpretation of the facts as reported.

Yellow fever in Africa has been confined to the West Coast mainly between Senegal and the mouth of the Congo; centering about Sierra Leone and the Gulf of Guinea.

The writer does not know, and cannot find out, anything about the trade relations between this section and Asia and Australia. He cannot then estimate the direct risk of the conveyance of yellow fever thereby. There is probably very little direct trade, there seems no reason why there should be, and the time *en route* would be very long. He knows of no instance in which yellow fever was conveyed even in this direction, although it has been carried from this coast to St. Helena, Ascension, and Cape de Verde Islands, and the West Indies, and probably to Brazil. In the absence of more definite knowledge he is inclined to consider the direct risk of conveyance minimal or nil.

Nevertheless there is yellow fever in Africa, and apparently in permanence. It has been reported, after a rather long quiescence (Gouzien, 1922, and United States Public Health Reports, 1922 and 1923) since June, 1922, at a number of places between Gambia and the mouth of the Congo. It is in general recognized only when it appears among whites. Among them it has appeared at almost every port of this coast, and as far inland as they have formed settlements: as at Sekom-Sikoro last October—over 600 miles inland. Among negroes it is rarely recognized but occurs, probably mainly in childhood and youth. *Ægypti*, and other *Stegomyia* as well, are abundant everywhere in this region—the coast and coastal plain—and the negroes doubtless furnish the human hosts

needed to continue the infection. There seems no reason then why yellow fever should not have extended, carried by negroes in the stage of incubation, along the routes of travel as far as the presence of active *ægypti* allows. That it has not reached the east coast is apparently due to (1) the difficulty of communication; travel across the continent being on foot and until recently very rare; and (2) to the high and sparsely inhabited plateau which separates the coasts. The mean elevation of this is given as about 3,500 feet, and while this may not be above the possible breeding zone of the vector it is close to the limit, and the insect should be extremely rare or absent therein—even if it were well populated and breeding places provided. There are no closed vehicles making this trip which could convey infected or any other *ægypti*. Such men as had contracted yellow fever on the western side of the plateau would develop it *en route* and pass the stage infective to mosquitoes before they descended into the *ægypti*-infested lowlands to the east. The east coast has then in the past been well protected. Certainly we have no evidence that yellow fever has ever been conveyed thereto.

With only the same communication in the future (so rare and so long *en route*), the chance is that it would never be thus conveyed, but we cannot believe that these travel conditions will continue. When railroad communication across the continent in this region is established—indeed it need only be across the plateau—men infected west of the plateau can pass it in the stage of incubation and develop yellow fever in the infectable (*ægypti* infested) territory to the east of it.

Given the indefinite continuation of yellow fever in West Africa this seems to me inevitable. The east coast once infected, the risk to India, and thence to all of south and south-east Asia, would be great—permanently infected, very great; I think, inescapable. And, unless prevented by sanitary measures, it would probably be permanently infected.

The distances of Asia from East Africa and the islands which would become infected with it are great—from 2,000 to 3,000 miles—and protection against ship-borne infection from that distance can be made very effective. Yet it (the protection given by maritime quarantine) is confessedly not perfect; the chance of failure increasing with the length of time it must be kept up. We must expect, then, that infection would occasionally slip by if the infection of East Africa were general, and lasted indefinitely. The direct risk to eastern countries from Africa now may indeed be nil, but the indirect risk through the future infection of the east coast seems to me very real and very grave, and, unless this is prevented by sanitary measures, inevitable.

These measures, too—if possible, the elimination of yellow fever from West Africa, but at least such elimination and control that it would not be carried across to the East Coast—should be made effective before any

railroad across Africa connecting the infected and clean, but infectable, regions on the east is built. Indeed, commercial reasons to the contrary, the building of such a road should be delayed until the fever is eliminated about its western end. It may even be that the ravages of African *trypanosomiasis* have been paid for by delaying progress in Central Africa.

The risk of the introduction of yellow fever into Asia from America is, as we have seen, small. Small as it is, it is too great. It should be none. In 1916, the writer said: "The result of the introduction of yellow fever into the dense, imperfectly educated, and very conservative populations of Asia, where *Calopus ægypti* is abundant, would be so stupendous a calamity that one can scarcely conceive of a risk of such a thing small enough to be allowable." He was pressing for its reduction to zero, so far as the Americas were concerned, by the elimination of all foci from them. He cannot understand, then, the absence of effort to guard against this—future indeed, but apparently inevitable—threat of infection from Africa. Guiteras called attention to it some years ago, and Malcolm Watson regards it nearly as gravely as does the writer. Had he seen as much yellow fever, he would do so equally. Neither of these sanitarians are alarmists, and the writer has been criticized for undue optimism as regards yellow fever. Yet is he persuaded that if yellow fever be introduced into south-eastern Asia, under the conditions which now obtain, we would look back to the early plague years there as years of comparative healthfulness.

BIBLIOGRAPHY.

Fielding, J. W.—Notes on the Bionomics of *Stegomyia fasc.* (Fabr.). *Ann. Trop. Med. and Parisitology,* Liverpool (1919-20). Vol. 13, p. 259.

Flu. P. C.—Tests of the Duration of Life of *Stegomyia fasciata* in low Temperatures. *Mededeel v. d. Burgerl. Geneesk. Dienst en Nederl. Indie,* 1920, p. 99.

Gouzien.—Pres. du Cons. Sup. de Santé des Colonies. La Fievre Jaune en Afrique Occidentale en 1922. *Office International d'Hygiene Publique.* Bulletin Mensuel, T. XIV.—Fascicule, 12, p. 1516.

United States Public Health Reports.—August, 1922; May, 1923.

Taylor, F. H. (1919).—Contributions to a Knowledge of Australian *Cullicidae.* Australian Institute of Trop. Medicine (1917): Reports of Mosquito Survey of Queensland, Coastal Towns (two reports): *Op. Cit.* Reprint from Quarantine Service Publications, 1916.

Edwards, F. W. (1909).—Revised Key to African *Culicidae: Bulletin of Entomological Research.* A number of papers on the same general subject, but in India and South China. Same Journal. 1920, 1921, and 1922.

McFie and Ingram (1921).—*Culicidae* in West Africa, &c. Same Journal.
